# The effects of three hamstring programmes on strength and sprinting performance in female footballers: A randomised controlled trial

**DOI:** 10.1371/journal.pone.0342529

**Published:** 2026-02-24

**Authors:** Enda Whyte, Siobhán O’Connor, Aaron Connolly, Eve Hession, Jonathan Kennedy, Bernard O’Boyle, Joshua Thorp, Sam Timmons, Aoife Burke

**Affiliations:** 1 School of Health and Human Performance, Dublin City University, Dublin, Ireland; 2 Centre for Injury Prevention and Performance, Dublin, Ireland; Manchester Metropolitan University Faculty of Health and Education, UNITED KINGDOM OF GREAT BRITAIN AND NORTHERN IRELAND

## Abstract

**Objective:**

Hamstring strain injuries (HSIs) frequently occur in female football, with sprinting being a common mechanism of injury. During sprinting, the considerable strength requirements of the knee flexors and hip extensors indicate their importance in performance and may partially explain the high incidence of HSIs. This study examined the effectiveness of 3 interventions on hip extensor and knee flexor strength and sprint performance in female footballers using a randomised controlled study design (ClinicalTrails.gov: NCT0573327).

**Methods:**

Thirty-five healthy young female footballers (age = 20.9 ± 1.3 years, stature = 167.8 ± 5.4 cm, body mass = 66.8 ± 8.4 kg) from Dublin City University were randomized to Nordic hamstring exercise (NHE), single leg hamstring bridge (SLHB) or razor hamstring curl (RHC) interventions. A two-way repeated measures analysis of variance determined main effects of time (pre vs post), group (NHE vs SLHB vs RHC) and interaction effects on normally distributed peak torque variables of the dominant and non-dominant limbs (isometric knee flexor at 30° knee flexion, eccentric knee flexor during the NHE and isometric hip extensor at 0° and 30° hip flexion) and sprint variables (0-5m, 0-15m, 0-30m sprint times, maximal horizontal force production, theoretical velocity and horizontal power output). Non-normally distributed data (eccentric knee torque during the NHE) were investigated using Wilcoxon signed rank (time main effects) and Kruskal Wallis (group main effects) tests.

**Results:**

No interaction or group effects were observed for strength measures (*p* = 0.44–0.96, ɳ^2^=>0.01–0.05). There was a significant time effect for isometric knee flexor peak toque (*p <* 0.001;ɳ^2^ = 0.60–0.62) and knee flexor peak toque during NHE testing (*p =* 0.008–0.014;*r* = 0.29–0.32), and isometric hip extensor peak torque at 0° (*p <* 0.001;ɳ^2^ = 0.59–0.58) and 30° (*p <* 0.001–0.011;ɳ^2^ = 0.19–0.32). No interaction or main effects for sprint variables (*p* = 0.093–0.957, ɳ^2^ =<0.01–0.09) were observed.

**Conclusion:**

Participants in all 3 intervention groups demonstrated strength increases measurements after 4 weeks. Interventions of longer duration or different components should be considered for targeting improvements in sprint performance.

## Introduction

Hamstring strain injuries (HSIs) lead to a high injury burden in male and female football with HSIs leading to 66 days and 57 absent per 1000 hours in collegiate female and elite male Gaelic football respectively [1,2]. Approximately 60% of HSIs occur during high speed running [3]. It is theorised that HSIs occur during terminal swing phase when the hamstrings lengthen actively [4] as the hamstrings experience greater peak lengths and lengthening velocities. During the swing phase, the hamstrings and gluteals produce hip extensor and knee flexor moments, acting in a spring like fashion to slow down the forward momentum at the hip and knee [5], with the combination of hamstrings strength and surface electromyography (EMG) activity during late swing phase significantly contributing to horizontal ground reaction force production [6]. The large biomechanical demands on the hamstring and gluteal muscles indicate their importance in high speed running and performance may, at least partially, explain the observed propensity for hamstring injury during high speed running.

Numerous risk factors have been identified including hamstring specific issues such as deficits in isometric, [7] eccentric, [8,9] and endurance [10] strength. Proximal risk factors have also been found; greater gluteal and trunk strength and endurance [11] have been observed in those who did not sustain a HSI compared with those who did, while lower isokinetic concentric hip extensor strength was observed in those who subsequently sustained a HSI [9]. In terms of sprinting mechanics, low horizontal force production during in-season testing increased HSI risk in footballers [12]. Consequently, investigating the effects of different interventions on a range of potential risk factors and performance outcomes are needed to refine injury prevention programmes [13].

The Nordic hamstring exercise (NHE) is a knee dominant hamstring exercise found to increase hamstring eccentric strength, improve sprint times and sprint mechanics [13]. Previous studies have demonstrated that short duration NHE interventions of 4 weeks increase hamstring strength [14–16], fascicle length [14], and can improve sprint times [13]. However, research is required to see if other exercises are more effective than NHE interventions [13]. Hip dominant hamstring muscle strengthening exercises, such as the single leg hamstring bridge (SLHB) leads to greater surface hamstring and gluteal surface EMG activity compared to the NHE [17], while the razor hamstring curl (RHC) leads to greater peak eccentric knee flexor force that the NHE. [18] However, the effect of these hip dominant exercises on HSI risk factors and sprint performance is not well understood. In addition, the majority of studies investigating the effect of hamstring interventions have been conducted on males, with a specific need to investigate the effects in females. [13,19] Therefore, the research question for this study was to determine the effects of three different hamstring strengthening exercises (NHE, SLHB and RHC) on dominant and non-dominant knee flexor and hip extensor strength measures and sprint performance in female footballers. The objectives were to assess the isometric and eccentric knee flexor and isometric hip extensor strength (Newton metres per kilogramme body mass) and sprint performance (split times, maximal horizontal force production [F_H0_], theoretical velocity [V_0_], and horizontal power output [P_max_]) before and after a 4 week hamstring strengthening intervention and to compare the effects of the different interventions on the strength and sprint variables. We hypothesised that

1. Participants in the 3 intervention groups would demonstrate similar improvements in knee flexor strength measures
2. Participants in the hip dominant interventions (SLHB and RHC) would demonstrate greater hip extensor gains compared to the NHE
3. Participants in the 3 intervention groups would demonstrate improvements in sprint performance.

## Methods

### Study design

We conducted a randomised controlled trial on female Gaelic footballers from Dublin City University academy to investigate the effects of a 4-week NHE, SLHB or RHC exercise programme on knee flexor and hip extensor strength and measures of sprint mechanics and time. The CONSORT 2010 guidelines for reporting a randomised controlled trial (S1 File) were complied with and the trial was registered (ClinicalTrials.gov identifier: NCT0573327). An a priori power analysis using G*Power 4.0 (ANOVA: repeated measures, within–between interaction; 3 groups × 5 timepoints) with f = 0.40, α = 0.05, power = 0.80, r = 0.80, and ε = 0.75 indicated a required total sample size of 36 participants. The effect size was informed by meta-analysis standardised mean differences for eccentric hamstring-strength adaptations (0.68–1.11) [20,21]. Volunteers were randomised to the NHE, SLHB or RHC groups by the lead author (EW) using a 6-block randomisation pattern (www.sealedenvlope.com). The NHE group were considered to be the control group given that NHE interventions have been repeatedly found to increase eccentric peak knee strength [13]. The participants or the authors who delivered the intervention or assessed the outcomes were not blinded (AC, EH, ST, BB, JK, JT). Evaluations were performed before and after the NHE, SLB and RHC programmes in the Athletic Therapy laboratory of Dublin City University.

### Participants

All female collegiate Gaelic footballers (n = 342) at Dublin City University were invited to participate between the 20^th^ of January and 10^th^ of February, 2023. Inclusion criteria were that participants must 18 years or older, free from hamstring injury in the last 6 months, and currently participating in Gaelic football at a varsity level on at least 3 occasions per week. Exclusion criteria included a history of hip or knee injury within the last 3 months, a history of anterior cruciate ligament rupture or involvement in a hamstring strengthening programme within 3 months of the start of the study. The study was approved by the Dublin City University Research Ethics Committee (DCUREC 2022_ATT_EW_15) and written informed consent was provided by all participants. Participants attended two post-intervention sessions between the 27^th^ of March and 5^th^ of April 2023.

A familiarisation session and two testing sessions were conducted prior to the intervention from the 13^th^ to 24^th^ of February 2023, with each session separated by 3–5 days. Testing procedures and relevant hamstring strengthening exercises were explained and demonstrated. We provided participants with the opportunity to ask questions and practice the testing and exercises with feedback from the investigators where necessary. Participants completed general health and injury questionnaires, and we recorded their body height, mass, chronological age and leg lengths during the familiarization session. Participants were instructed not to commence the exercise programme until the intervention period began. The pre- and post-testing sessions were completed within 6 days of the intervention. Strength assessments were completed on the first day of testing with sprinting performance on the second day.

### Strength assessment

We conducted isometric strength assessment of the knee flexors and hip extensors and eccentric assessment of knee flexor strength during the NHE exercise on participant’s dominant and non-dominant limbs. Isometric assessments were conducted using a microFET^®^2 Digital Handheld Dynamometer (HHD) (Hoggan Scientific, Salt Lake City, UT) with a sampling frequency of 60 Hz and limb dominance was defined by which leg the participant would prefer to kick a ball with [22]. Participants completed a standardised warm-up on a stationary bicycle ergometer between 80 and 100 rpm for 10 minutes followed by two practice repetitions of each at 60% and 80% of maximal effort. Rest intervals of 10 seconds were given between each practice and a 20 second interval between the final practice and maximal test effort. A 1 minute rest was given between each maximal effort test. They were directed to use maximum effort during the maximal effort test and instructed to “Go ahead-push-push-push-push-push and relax”, for each contraction. Each contraction was isometrically resisted by the examiner for approximately 3 seconds during which the tester monitored the HHD display to ensure the reading had plateaued. The highest score of three trials was recorded. We conducted a preliminary intra-tester reliability study that demonstrated a high level of reliability for the strength assessments (ICC 0.89–0.96) (see S2 File).

**Fig 1:**
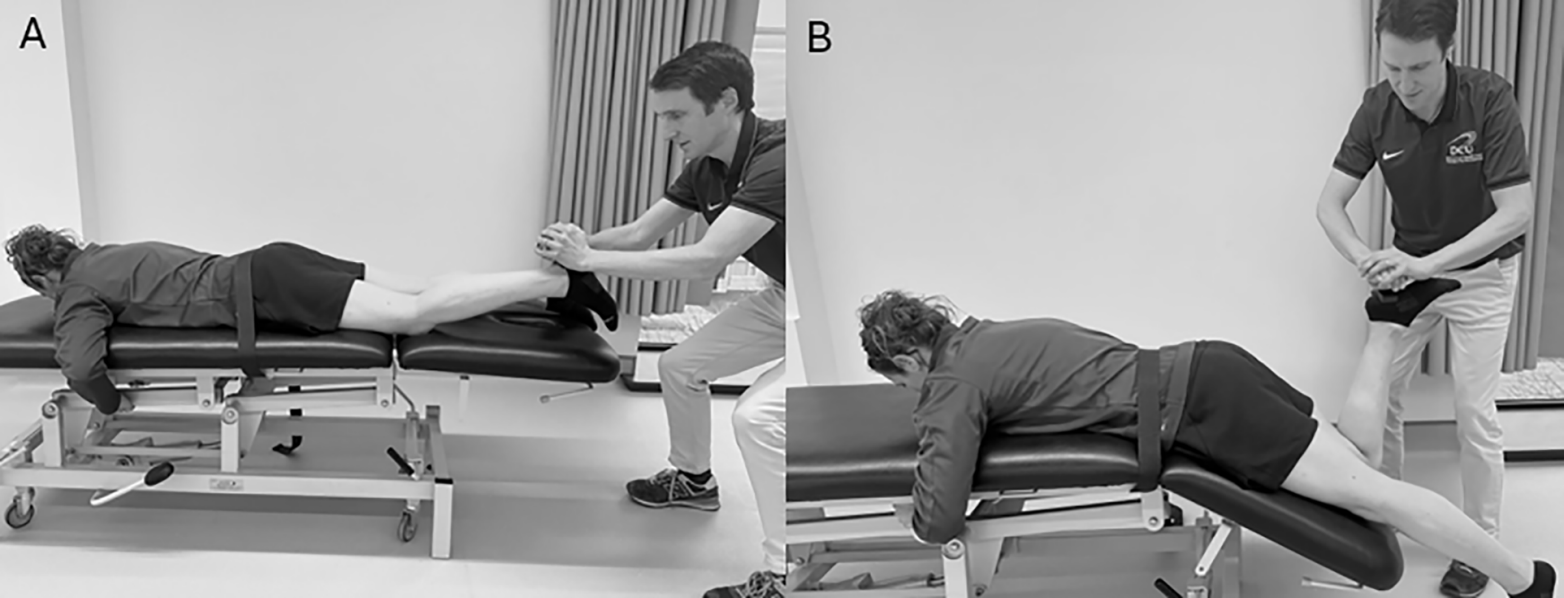
Positions for Isometric Strength Testing: A Isometric Knee Flexor Strength, B Isometric Hip.

Knee flexor strength was assessed as previously described [23] with the hips in neutral and the knee flexed to 30°, confirmed with use of a goniometer. A ‘break force’ was obtained by pulling down the athlete’s heel to the plinth once the examiner felt the force had peaked and confirmed by monitoring the HHD device (Fig 1). Knee flexor moment arm was by measuring the distance from the lateral femoral epicondyle, which has been shown to represent axis of rotation of the knee joint [24], to 5cm proximal to the lateral malleolus, where the HHD was positioned. Isometric hip extensor testing at 0° hip flexion was completed in prone. A section of the plinth was adjusted to 30° below horizontal so that in prone, participants’ hips were positioned in 30° flexion and the knee flexed to approximately 120° knee flexion (Fig 1). The HHD was positioned on the plantar surface of the calcaneus, directly over the lateral femoral condyle, and the participant was instructed to push their heel up towards the ceiling with the action resisted by the researcher for both tests. Hip extensor moment arm was calculated by measuring the distance from the superior tip of the greater trochanter to the position of the HHD [25], which is this study was directly over the lateral femoral epicondyle. Relative knee flexor and hip extensor peak torque (Nmkg^-1^) was recorded for each participant [13]. Participants were given a 15-minute break between each set of isometric tests set to minimise the effects of fatigue. Following this, participants were given a 60-minute break before repeating the standardised warm up and completing the NHE knee flexor strength assessment.

All NHE assessments were performed on the ‘Hamstring Solo Elite’ (ND Sports Performance, Kilkenny, Ireland) at a sampling frequency of 50 Hz. The device has two separate rigid heel fixators positioned 45 cm above the ground to allow for full knee extension during the NHE, cushioning for the shank which allowed partial glide of the patellae, with the shanks supported at an angle of 30° from horizontal. Participants completed three practice NHE repetitions at 50%, 75% and 95% of subjectively rated maximal effort, followed by a 1-minute rest before three maximal repetitions. Participants were requested to maintain their arms across their chest and to lean forwards from the knees, lower their body as slowly as possible (aiming for 4−6 seconds using a metronome at 60 bpm) through as far a range as possible. To facilitate maximal effort, participants were given real time feedback during the NHE. If participants moved into hip flexion or lumbar lordosis during the test, that repetition was excluded, corrective feedback was given and repeated. We assessed if a participant needed additional resistance by positioning a pole at the participant’s knee at an angle of 20° to assess if the participant was able to control the movement into the final 10−20° of motion [26]. No participants were able to achieve this and therefore additional resistance was not required during testing. Relative peak eccentric torque during NHE testing was recorded (Nmkg^-1^) [13].

## Sprint

We assessed sprint performance as described by Ishoi et al., 2019 [27] following a standardised and supervised warm-up (FIFA11+) with progressive running and sprinting which lasted approximately 20 minutes. We positioned timing gates with single-beam photoelectric cell systems (Brower TCi Timing Systems, Salt Lake City, Utah, USA) at 0m, 5m, 10m and 30m, 2 metres apart from each other. Participants started in a 3-point start position with one hand on a line representing 0 metres. The first speed gate was placed at ground level, with the timing gate beam positioned immediately in front of the toes of the back leg. The remaining speed gates were positioned at a height of 100 cm. Each participant performed three maximum effort 30-metre sprints with 2-minutes rest between attempts to minimize the effects of fatigue. The start of the sprint was calculated by using a high speed camera (Canon Legria HF R706, Ota, Tokyo, Japan) to measure the time difference between the initial sprint movement and breaking of the first timing gate. This time was added to sprint and split times to make the corrected times [27]. The fastest corrected time recorded and the corresponding three corrected split times were used to calculate the mechanical sprint variables (maximal horizontal force production [F_H0_], theoretical velocity [V_0_], and horizontal power output [P_max_]) using a recognised calculation process [28,29], with F_H0_, and P_max_ normalised to body mass. Based on the best sprint performance, 0–5 m, 0-10m and 0-30m split times (s) and mechanical sprint variables (F_H0_, V_0_, P_max_) were recorded.

## Interventions

**Fig 2:**
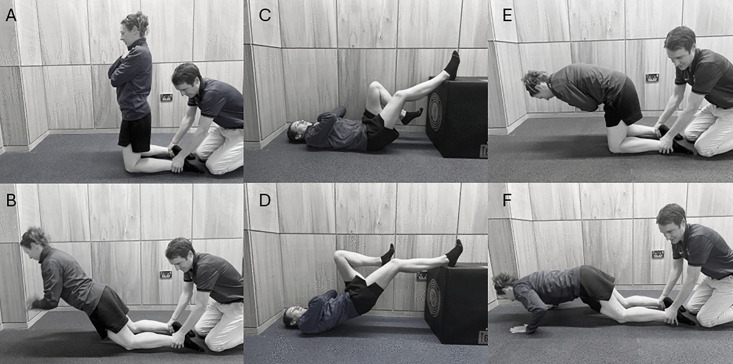
Exercise Interventions: A and B start and end position of the Nordic Hamstring Exercise; C and D start and end position of the single leg hamstring bridge exercise; E and F start and end position of the razor hamstring curl.

The NHE [30], RHC [30] and SLHB [10] interventions were completed by participants (see Fig 2, S3 File). If participants were able to control the movement over the last 20° of knee flexion in the NHE or to achieve almost full knee and hip extension for the RHC, additional weight in 2.5 kg increments were added to the exercise to ensure the intensity of the exercise [30], however, this was not necessary.

All participants completed a supervised 4-week intervention (Table 1) in the Dublin City University’s Athletic Therapy laboratory. A previous study demonstrated that the number of sets and repetitions and duration of the NHE programme significantly increased eccentric knee flexor peak strength [15], Sessions were supervised by final year athletic therapy and training students who were trained in the interventions by EW, a certified athletic therapist and physiotherapist with more than 20 years’ sports medicine experience. We gave participants a recovery time of 10 seconds and 3 minutes between each repetition and set of the NHE and RHC exercises respectively. Participants were given a 30 second recovery between limbs and 3 minutes between sets of the SLHB exercise. Management agreed not to hold lower limb resistance sessions for 48 hours after each intervention session. If a participant was unable to attend a session, they were required to provide video evidence of completion of the appropriate number of exercises within 48 hours of the missed session. Participants had to complete a minimum of 80% of the intervention sessions to be included in the post-intervention assessments, which in practice meant they had to attend for a minimum of seven sessions. Participants were instructed not to complete additional training outside of the interventions and their normal team training programmes and match participation.

**Table 1:** Details of the Nordic Hamstring, Single Leg Hamstring Bridge and Razor Hamstring Curl exercise programmes.

Week	Frequency	Sets	Repetitions	Total repetitions per week*
1	2	2	6	24
2	2	3	6	36
3	2	4	8	64
4	2	4	10	80

* The number of repetitions are for individual legs for the single leg bridge exercise.

## Statistical analysis

Statistical analysis were performed using SPSS (IBM Corp. Released 2021. IBM SPSS Statistics for Windows, version 30.0. Armonk, NY,:IBM Corp). We used the Shapiro-Wilk test to examine for normality and conducted a two-way repeated mixed between-within analysis of variance on all data, (with the exception of peak knee flexor torque during the NHE) to examine the interaction and main effects of time (pre vs. post) and group (NHE vs, RC, vs. SLHB) for strength measures of the dominant and non-dominant limbs individually (isometric peak hip extensor torque at 0° and 30° hip flexion, isometric peak knee extensor torque at 30° knee flexion) and performance measures (0-5m, 0-15m, 0−30 m sprint times, F_H0_, V_0_, Pmax). As the peak knee flexor torque during NHE data was not normally distributed, we used a Wilcoxon signed rank test to examine the main effect for time and the Kruskal Wallis test to check for main effect of group. Partial eta-squared effect sizes (η^2^) for interaction effects were classified as small 0.01–0.059; medium 0.06–0.14; and large ≥0.14. [31] If significant group main effects were observed, a Bonferroni post hoc adjustments was made for three comparisons (NHE pre – NHE post, RHC pre – RHC post, and SLHB pre – SLHB post) with a resulting *p* value of 0.017. Percentage changes after the intervention were calculated for the variable.

## Results

**Fig 3:**
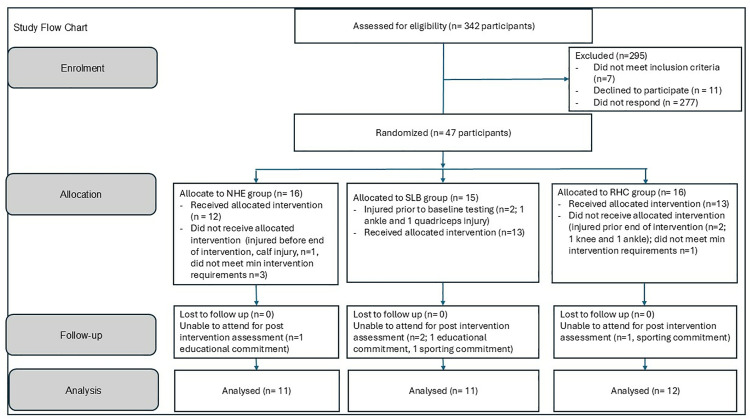
Study Flow Chart.

Between January and February 2023, 342 female athletes from a Dublin City University’s Gaelic football academy were assessed for eligibility. Please see Fig 3 for detailed participant flow which left 12, 11 and 12 in the NHE, SLHB and RHC groups respectively (see Table 2 for participant characteristics). One participant in the NHE and RHC groups had missing post intervention sprint data and isometric strength measures respectively. Study data and statistical output available on available at https://osf.io/7632p/.

**Table 2:** Participant Characteristics.

Group	Age (years)	Stature (cm)	Body Mass (kg)
**NHE**	21.1 (± 1.5)	168.2 (± 6.3)	64.9 (± 7.7)
**SLHB**	21.3 (± 1.1)	168.4 (± 5.5)	65.5 (± 6.8)
**RHC**	20.3 (± 1.2)	166.7 (± 4.5)	70.0 (± 9.9)

NHE – Nordic hamstring exercise, SLHB – Single leg hamstring bridge, RHC – Razor hamstring curl.

### Strength measurements

No group (NHE *vs*. SLHB *vs.* RHC) by time (pre *vs*. post) interaction effects were observed for isometric knee peak flexor and isometric hip extensor peak torque at 0° and 30° hip flexion for dominant and non-dominant legs (*p* = 0.44–0.96, ɳ^2^=>0.01–0.05) (see Table 3). There was a main effect for time for dominant and non-dominant leg isometric knee flexor peak torque with large effect sizes (*p <* 0.001;ɳ^2^ = 0.60–0.62), isometric hip extensor peak torque at 0° large effect sizes (*p <* 0.001;ɳ^2^ = 0.59–0.58) and isometric hip extensor peak torque at 30° with large effect sizes (*p <* 0.001–0.011;ɳ^2^ = 0.19–0.32). There were also significant main effects for time for peak knee flexor torque during the NHE for dominant and non-dominant limbs with small to moderate effect sizes (*p =* 0.008–0.014;*r* = 0.29–0.32). A significant main effect for group for dominant leg isometric hip extensor peak torque at 0° was observed (*p* = 0.039,ɳ^2^ = 0.19) and 30° (*p* = 0.046,ɳ^2^ = 0.18). A post hoc Bonferroni adjusted alpha level of 0.17 revealed no significant differences between the groups for dominant limb, isometric hip extensor peak torque at 0° (NHE *vs.* RHC *p* = 0.038; NHE *vs*. SLHB *p* = 0.326; RHC *vs.* SLHB *p* = 1.00) or 30° (NHE *vs.* RHC *p* = 0.054; NHE *vs*. SLHB *p* = 1.00; RHC *vs.* SLHB *p* = 0.187). There were no other main effects for group for the remaining strength measures.

**Table 3:** Mean and SDs for dependent measures and effect sizes of main and interaction effects for strength measures.

		Group									Main Effect					
		Nordic Hamstring exercise (n = 12)			Single Leg Hamstring Bridge (n = 11)			Razor Hamstring Curl (n = 12)			Time		Group		Interaction Effect	
		Pre	Post		Pre	Post		Pre	Post		*p*	η^2^	*p*	η^2^	*p*	η^2^
Leg		Mean (SD)	Mean (SD)	%	Mean (SD)	Mean (SD)	%	Mean (SD)	Mean (SD)	%						
Isometric knee flexor peak torque (Nm/kg)	ND	1.37 (0.22)	1.56 (0.26)	13.6	1.38 (0.28)	1.54 (0.20)	11.0	1.20 (0.19)	1.37 (0.21)	14.6	<0.001	0.62	0.129	0.13	0.842	0.01
	D	1.45 (0.25)	1.65 (0.26)	14.0	1.40 (0.28)	1.55 (0.23)	10.9	1.26 (0.28)	1.48 (0.29)	17.4	<0.001	0.60	0.255	0.09	0.620	0.03
Peak Knee Flexor torque during NHE** (Nm/kg)	ND	1.07 (0.38)	1.21 (0.41)	12.9	0.99 (0.22)	1.01 (0.27)	1.9	1.03 (0.32)	1.15 (0.36)	11.6	0.008	0.32	0.428	0.00		
	D	1.20 (0.38)	1.41 (0.40)	17.5	1.10 (0.24)	1.10 (0.27)	0.1	1.15 (0.36)	1.36 (0.41)	18.0	0.014	0.29	0.278	0.01		
Isometric hip extensor peak torque 0° (Nm/kg)	ND	1.34 (0.18)	1.63 (0.22)	20.7	1.35 (0.31)	1.52 (0.22)	13.0	1.26 (0.22)	1.48 (0.22)	17.0	<0.001	0.59	0.394	0.06	0.437	0.05
	D	1.50 (0.18)	1.72 (0.20)	14.2	1.35 (0.31)	1.60 (0.24)	17.9	1.28 (0.20)	1.51 (0.23)	17.8	<0.001	0.48	0.039*	0.19	0.963	0.00
Isometric hip extensor peak torque 30° (Nm/kg)	ND	2.18 (0.44)	2.44 (0.38)	11.9	2.15 (0.50)	2.35 (0.29)	9.1	1.94 (0.40)	2.03 (0.27)	4.6	0.011	0.19	0.072	0.16	0.578	0.04
	D	2.21 (0.36)	2.45 (0.35)	11.2	2.14 (0.46)	2.36 (0.40)	10.3	1.88 (0.41)	2.06 (0.30)	9.4	<0.001	0.32	0.046*	0.18	0.872	0.01

D = dominant limb; ND = non dominant limb; Nm/kg = newton metres per kilogramme body mass;

*Post hoc analysis -difference between NHE and RHC p = 0.038, 0.054 – not significant when adjusted for Bonferroni (p < 0.017).

** Data was not symmetrical. Wilcoxon used to determine main effect of time, Kruskal Wallis test used to determine main effect of group.

### Sprint performance measurements

In relation to the 0–5, 0–10 and 0–30 sprint times, there were no observed interaction effects (*p* = 0.479–0.682, ɳ^2^ = 0.02–0.05), main effects for time (*p* = 0.093–0.199, ɳ^2^ = 0.02–0.05) or main effects for group (*p* = 0.330–0.717, ɳ^2^ = 0.05–0.09) (see Table 4). Similarly, for sprint biomechanical variables (F_H0_, V_0_, Pmax), there were no observed interaction effects (*p* = 0.600–0.957, ɳ^2^=< 0.01–0.03), main effects for time (*p* = 0.468–0.738, ɳ^2^=<0.01–0.02) or for group (*p* = 0.647–0.902, ɳ^2^ = 0.02–0.06).

**Table 4:** Mean and SDs for dependent measures and effect sizes of main and interaction effects for sprint performance.

	Group									Main Effect					
	Nordic Hamstring exercise (n = 12)			Single Leg Hamstring Bridge (n = 11)			Razor Hamstring Curl (n = 12)			Time		Group		Interaction Effect	
	Pre	Post		Pre	Post		Pre	Post		*p*	η^2^	*p*	η^2^	*p*	η^2^
Leg	Mean (SD)	Mean (SD)	%	Mean (SD)	Mean (SD)	%	Mean (SD)	Mean (SD)	%						
5 metre sprint (s)	1.28 (0.07)	1.30 (0.08)	1.6	1.30 (0.09)	1.31 (0.11)	1.0	1.32 (0.09)	1.32 (0.09)	0.1	0.153	0.07	0.717	0.02	0.575	0.04
10 metre sprint (s)	2.15 (0.11)	2.14 (0.10)	−0.6	2.21 (0.13)	2.19 (0.13)	−0.8	2.23 (0.11)	2.19 (1.0)	−1.8	0.093	0.09	0.330	0.07	0.682	0.02
30 metre sprint (s)	5.15 (0.30)	5.14 (0.33)	−0.1	5.29 (0.34)	5.27 (0.37)	−0.3	5.35 (0.36)	5.28 (0.39)	−1.2	0.199	0.05	0.472	0.05	0.479	0.05
F_H0_ (N/kg)	10.03 (1.26)	9.82 (0.90)	−2.2	9.67 (1.40)	9.50 (1.44)	−1.7	9.61 (1.47)	9.54 (0.86)	−0.7	0.468	0.02	0.707	0.02	0.957	0.00
V_0_ (m/s)	6.92 (0.52)	6.95 (0.55)	0.4	6.83 (0.60)	6.78 (0.58)	−0.9	6.66 (0.70)	6.75 (0.67)	1.3	0.738	0.00	0.647	0.03	0.600	0.03
P_max_ (W/kg)	17.33 (2.35)	17.08 (2.3)	−1.4	16.42 (2.38)	16.10 (2.76)	−1.9	15.91 (2.43)	16.11 (2.26)	1.2	0.708	0.01	0.902	0.06	0.776	0.02

F_HO_ = maximal horizontal force production; V_0_ = maximal theoretical velocity; P_max_ = maximal horizontal power output; s = seconds; N/kg = newtons per kilogramme body mass; m/s = metres per second; W/kg = watts per kilogramme body mass.

## Discussion

This study aimed to investigate the effect of 4-week hamstring strengthening programmes (NHE, SLHB and RHC) on knee flexor and hip extensor strength and sprint performance. We hypothesised that the participants in the 3 intervention groups would demonstrate similar improvements in knee flexor strength measures, those in the hip dominant interventions (SLHB and RHC) would demonstrate greater hip extensor strength gains compared with the NHE and all three groups demonstrate improvements in sprint performance. The results of the study supported the first hypothesis as the participants in the three groups demonstrated increased post intervention knee flexor isometric and eccentric strength with large effect sizes. The results did not support our second hypothesis as there was no statistical difference in hip extensor strength improvements between programmes. Our final hypothesis is also rejected as the programmes did not influence sprint times or biomechanical measures.

The 4-week hamstring strengthening programmes resulted in increases in isometric and eccentric knee flexor strength with strong effects, supporting similar findings in a male and female cohort after a 4 week intervention [14]. A previous female specific study found that an NHE intervention required at least 6 weeks to increase eccentric hamstring strength significantly [32]. This is the first study, to our knowledge, that has demonstrated the effectiveness of a shorter duration NHE programme and reported the effects of the RHC and SLHB programmes in female athletes. As isometric [7] and eccentric [8] knee flexor deficits have been identified as risk factors for subsequent HSI, the results would suggest that a 4-week programme of different hamstring strengthening programmes may be used to address deficits identified in female athletes. There were no group main effects for eccentric strength between the interventions despite NHE and RHC interventions resulting in greater percentage changes with moderate effect sizes in strength in the non-dominant and dominant limbs compared with the SLHB intervention (NHE:12.6 and 17.5%; RHC:11.8 and 18.0%; SLHB: 1.9 and 0.1%). The NHE and RHC have been described as maximal effort exercises as they require participants to eccentrically control knee extension (NHE) and hip and knee extension (RHC) to the point of failure during each exercise [30], generating high surface EMG activity levels [33,34]. Each individual SLHB exercise in the current study was not a maximal effort exercise which may generate a lower strengthening stimulus. Studies investigating the effect of short duration training interventions of 4 and 6 week comparing maximal versus submaximal training in untrained males found greater strength gains in the maximal training groups [35,36]. As it has been found that found that completing the SLHB at higher speeds and using higher platforms results in greater hamstring surface EMG activity [37], further research examining the strengthening effect of different SLHB effort exercises in trained, female participants is required.

The 4-week strengthening programmes resulted in increases in isometric hip extensor strength at 0° and 30° hip flexion with strong effects bilaterally. Carmichael et al., [38] found that a 6-week isometric hip strengthening exercise programme resulted in a 12% increase in isometric hip extensor strength at 0° hip flexion in recreationally active males, similar to the values reported in the current study (13–20%). There was also a group main effect observed for dominant limbs for isometric hip extensor strength at 0° and 30° hip flexion although post hoc analysis did not find a significant difference between the groups. Given that greater trunk and gluteal isometric strength reduces HSI risk, [11] and lower concentric hip extensor strength has been identified as a risk factor for HSI, [9] increasing hip extensor strength is an important consideration in HSI prevention and rehabilitation. A 4-week programme of the NHE, SLHB or RHC may be appropriate to achieve this goal in female footballers, with the NHE potentially being more practically impactful. Prospective studies comparing the effects of the NHE and RHC on HSI rates in female footballers is required to fully understand the potential benefits of these exercises.

The interventions did not affect sprint times or mechanical properties despite the increases in hip extensor and knee flexor strength observed (see Table 4). This contrasts with a meta-analysis demonstrating a significant NHE-induced improvement on sprint times when combined over 5, 10 and 20m distances [13]. However, a minority of the participants of the total sample size were females (24/165) with only one study with a cohort solely of females [39]. Chaabone et al. [39] found that an 8-week NHE programme significantly improved 5, 10 and 20m sprint times in adolescent female handball players while, Amundsen et al. [32] did not observe any improvements in sprint times (5, 10, 20, 30 and 40m) in highly trained, adult female soccer players following high and low volume NHE programmes over 10 weeks. The differences between the results of the studies could, at least partially, be explained by the fact that the effects of NHE training on sprint performance in well trained team sport players is less consistent and that a higher training intensity during the NHE may be needed in well trained athletes to see benefits [13]. It is possible that the adult players were at a higher training level than the adolescent population. Also, no significant correlations were found between knee flexor strength (isometric, isokinetic or eccentric during the NHE) and sprint times in elite female Gaelic footballers [40]. It may be that the hip extensors and knee flexors contribute to sprinting in a different manner in females compared with males, a pattern that has been observed in hamstring activity in lower intensity activities [41]. Also, the strength measures taken in the current study were isometric or slow eccentric which may not appropriately measure the contractile properties critical for sprint performance.

Although no statistically significant improvements in sprint performance were observed, Haugen and Buchheit [42] calculated that the smallest worthwhile change (SWC) for team sport players is ~ 1.5% for 5 m sprints and~1% for 10–40 m sprints based upon statistical considerations of a range of appropriately powered and described studies or a 20 cm increase in distance covered, based on the running speed improvement required to win a ball in football. We observed smallest worthwhile changes following the NHE intervention at 5 metres and the RHC at 10 and 30 metres. For the NHE, there was a decrease in performance of 1.6% (0.02 s) which corresponds to a decrease of approximately 4 cm. On the other hand, the RHC intervention resulted in improved performances of 1.8% (0.04 s) and 1.2% (0.07s) in the 10m and 30m sprint distances respectively, corresponding to approximately 18 cm and 40 cm improvements, which may be relevant in practice.

As there is a relationship between eccentric knee flexor strength combined with surface EMG activity and FH_0_ [6], we hypothesised that increased knee flexor strength post intervention would lead to improved sprint mechanics. However, this was not the case. Previous research examining the effects of hamstring strengthening programmes have reported inconsistent findings. A study on male Australian footballers found that a 14-week NHE programme resulted in improved 5m and 10m sprint times, but not FH_0_, although there was an increase in V_0_ [43]. Timmins et al., [26] also found that the relationship was not straightforward in a study examining the effects of a hip dominant exercise programme and the NHE in addition to sprint training. They found that the NHE group improved 5m sprint times after 16 weeks of the intervention period only while there were no changes in mechanical sprint profiles. On the other hand, there were significant increases in V_0_ in the flywheel group after 16 weeks, followed by a significant decrease after 39 weeks and an increase in increase in FH_0_ at end of the intervention. As the relationship between hip extensor and knee flexor strengthening programmes and mechanical profiles is not clear, future research should expand the range of strength variables, such as rate of torque development, [27] assessed in females to better investigate the relationship with sprinting performance.

There are several limitations to this study. Firstly, we did not include a group without a hamstring strengthening intervention to avoid the potential for an increased risk of injury in a group without a hamstring strengthening programme. Consequently, any changes observed could therefore be due to the football training undertaken. However, we consider this unlikely as 4 weeks of football training did not increase knee flexor strength in well trained soccer players [44]. Secondly, the NHE and RHC could be considered maximal effort exercises whereas the SLHB is not. This may have resulted in a lower training stimulus with the SLHB. The SLHB was performed as initially described as deficits in this measure has been previously shown to be associated with increased risk of HSI [10] and the effect of a SLHB intervention does not seem to have been investigated. Future research should investigate the effects of adjusting the SLHB exercise to increase the training stimulus. Also, although the number of participants that were initially randomised to the different groups was 47, the number of participants who completed the study was 35, which did not meet the sample size calculation. This will limit the generalisability of the study findings. In addition, we used clinician based resistance as opposed to externally fixed resistance. Both methods have been found to have high levels of reliability although differences in absolute strength measures have been found with some studies demonstrating greater strength scores using externally fixed resistance compared to clinician based resistance [45,46] and others finding the opposite [47,48]. It is important to consider the method of strength assessment when interpreting results from the current study. Finally, the strength measurements taken in this study were isometric and slow eccentric exercises. Although deficits in these measurements have been identified as potential risk factors for HSIs, [7,8] they may not assess muscular properties best associated with sprinting performance.

## Conclusion

Elite, female Gaelic footballers demonstrated increases in knee flexor and hip extensor strength following a 4-week hamstring strengthening programme of NHE, SLHB or RHC exercises with no statistical differences between the interventions. As there was no group without a strengthening programme, it is not clear if the improvements were due to the interventions or other factors, such as the regular training. There were no changes in sprint times or sprint mechanics. Longer duration interventions should be considered to determine their effect on sprint performance.

## Supporting information

S1 File The CONSORT 2010 guidelines for reporting a randomised controlled trial. (DOC)

S2 File Preliminary intra-tester reliability study of the strength assessments used in the study. (DOCX)

S3 File Details of the interventions used in the study. (DOCX)
