# Supplementary material for: The effects of three hamstring programmes on strength and sprinting performance in female footballers: A randomised controlled trial
Source: PLoS One. 2026 Feb 24;21(2):e0342529. doi: 10.1371/journal.pone.0342529 (PMC12931786; doi:10.1371/journal.pone.0342529)
Supplement: S2 File — (DOCX) [file pone.0342529.s002.docx]

**Supplementary Table 1 Intra rater Reliability of the Knee Flexor and Hip Extensor Strength Measurements**

| **Test** | **Leg** | **Time 1** | | **Time 2** | **Paired t Test** | | **ICC (CI 95%)** | | **SEM** | | **SEM%** | | **MDC** | | **MDC%** | |  |
| --- | --- | --- | --- | --- | --- | --- | --- | --- | --- | --- | --- | --- | --- | --- | --- | --- | --- |
|  |  | **Mean (SD)** | **Mean (SD)** | | | ***p* Value** | |  | |  | |  | |  | |  | |
| **Peak Isometric Knee flexor (N)** | ND | 282.53 (52.74) | 281.28 (49.22) | | | Ns | | 0.92 (0.77 -0.97) | | 14.2 | | 5.1 | | 39.3 | | 14.1 | |
|  | D | 277.49 (44.31) | 288.71 (42.54) | | | Ns | | 0.93 (0.80 - 0.98) | | 11.4 | | 4.0 | | 31.6 | | 11.9 | |
| **Peak Eccentric Force during the NHE (N)** | ND | 242.38 (78.18) | 246.41 (83.81) | | | Ns | | 0.96 (0.88 - 0.98) | | 15.93 | | 6.5 | | 44.2 | | 17.5 | |
|  | D | 257.64 (85.18) | 250.03 (76.70) | | | Ns | | 0.98 (0.93 - 0.99) | | 15.95 | | 6.3 | | 44.2 | | 16.6 | |
| **Peak Isometric Hip Extensor Strength at 0 (N)** | ND | 284.09 (38.84) | 278.07 (42.53) | | | Ns | | 0.96 (0.88 - 0.99) | | 8.03 | | 2.9 | | 22.3 | | 8.3 | |
|  | D | 295.53 (39.70) | 293.92 (45.23) | | | Ns | | 0.89 (0.69 - 0.97) | | 13.87 | | 4.7 | | 38.5 | | 13.4 | |
| **Peak Isometric Hip Extensor Strength at 30** | ND | 361.41 (40.57) | 368.88 (30.28) | | | Ns | | 0.90 (0.72 - 0.97) | | 11.12 | | 3.1 | | 30.8 | | 9.4 | |
|  | D | 384.92 (36.17) | 382.06 (28.38) | | | Ns | | 0.92 (0.76 - 0.97) | | 9.05 | | 2.4 | | 25.1 | | 6.5 | |

N newton, ICC intraclass correlation coefficient, CI confidence interval, SD standard deviation, SEM standard error of measurement, MDC minimal detectable change, % percentage, ns non-significant, ND non-dominant, D dominant.
