# Supplementary material for: The effects of three hamstring programmes on strength and sprinting performance in female footballers: A randomised controlled trial
Source: PLoS One. 2026 Feb 24;21(2):e0342529. doi: 10.1371/journal.pone.0342529 (PMC12931786; doi:10.1371/journal.pone.0342529)
Supplement: S3 File — (DOCX) [file pone.0342529.s003.docx]

**Exercise interventions description**

**Nordic Hamstring Exercise**

The NHE exercise was performed as previously described.^1^ Participants began in kneeling on a gym mat and kept their hips and spine in a neutral position. They were instructed to lower themselves down slowly in a controlled manner as far as they could towards the ground. When they lost the ability to control the movement, they were instructed to control the fall with their arms and touch their chest off the ground while keeping the hamstrings activated. They were instructed to immediately return to the starting position by pushing up with their hands, minimising the concentric activity of the hamstrings.

**Razor Hamstring Curl**

The RHC exercise was performed as previously described^1^. Participants began the exercise in the same starting position as the NHE. They then flexed their hips to approximately 90° so their torsos were approximately parallel to the floor. Participants were instructed to simultaneously extend their hips and knees, trying to keep their torsos parallel to the floor as far into hip and knee extension as possible and to continue until they could no longer control the motion and their chests or hands touched the floor. They then returned to the starting position by pushing up with their hands so that only the extension phase of the RHC was completed.

**Single Leg Hamstring Bridge**

For the SLHB exercise,^2^ participants lay supine with their arms across their chest. They placed one heel on a 60cm box, so their knees were in approximately 20° flexion. They then pushed down through their heel to lift their gluteals off the floor so that their hip was in neutral. They were then instructed to slowly lower their gluteals to the floor. The non-working leg was held in a stationary position of approximately 90° hip flexion and 145° knee flexion.

1. Pollard CW, Opar DA, Williams MD, Bourne MN, Timmins RG. Razor hamstring curl and Nordic hamstring exercise architectural adaptations: Impact of exercise selection and intensity. *Scand J Med Sci Sports*. 2019;29(5):706-715. doi:10.1111/sms.13381

2. Freckleton G, Cook J, Pizzari T. The predictive validity of a single leg bridge test for hamstring injuries in Australian Rules Football Players. *Br J Sports Med*. 2014;48(8):713-717. doi:10.1136/bjsports-2013-092356
